# Middle meningeal artery embolization without surgical evacuation for chronic subdural hematoma: a single-center experience of 209 cases

**DOI:** 10.3389/fneur.2023.1222131

**Published:** 2023-08-31

**Authors:** Atakan Orscelik, Yigit Can Senol, Cem Bilgin, Hassan Kobeissi, Santhosh Arul, Harry Cloft, Giuseppe Lanzino, David F. Kallmes, Waleed Brinjikji

**Affiliations:** ^1^Department of Radiology, Mayo Clinic, Rochester, MN, United States; ^2^Department of Neurologic Surgery, Mayo Clinic, Rochester, MN, United States

**Keywords:** middle meningeal artery (MMA), chronic subdual hematoma (CSDH), endovascular treatment, embolization procedures, polyvinyl alcohol (PVA)

## Abstract

**Background:**

Middle meningeal artery (MMA) embolization is a minimally invasive treatment option for new and recurrent chronic subdural hematomas (cSDH).

**Objective:**

To examine the safety and efficacy profile of MMA embolization without surgical evacuation for cSDH patients.

**Methods:**

A single-center retrospective study of patients with cSDHs treated by MMA embolization was undertaken. Patient demographics, hematoma characteristics, procedural details, and clinical and radiological outcomes were collected. The primary outcome was the need for retreatment, and the secondary outcomes were at least a 50% reduction in the maximum width of cSDH on the last CT imaging, complications, and an improvement in the modified Rankin scale (mRS) score. All results were presented as descriptive statistics.

**Results:**

A total of 209 MMA embolizations were successfully performed on 144 patients. Polyvinyl alcohol particles were the primary embolization agent in all procedures. Of the total of 206 cSDH, the median maximum width at pre-intervention and last follow-up were 12 and 3 mm, respectively, and the median reduction percentage was 77.5%, with a >50% improvement observed in 72.8% at the last follow-up imaging. A total of 13.8% of patients needed retreatment for recurrent, refractory, or symptomatic hematomas after embolization. The mRS score improved in 71 (49.3%) patients. Of 144 patients, 4 (2.8%) experienced complications related to the procedure, and 12 (8.4%) died during follow-up due to causes unrelated to the MMA embolization procedures.

**Conclusion:**

This study supports the fact that MMA embolization without surgical evacuation is a safe and effective minimally invasive option for the treatment of cSDHs.

## Introduction

Chronic subdural hematoma (cSDH) represents one of the most challenging neurosurgical diseases. The incidence of cSDH is highest among older adults, and its prevalence continues to rise with an aging population and the widespread use of antithrombotic medications (1–4). Large cSDHs presenting with a prominent mass effect or midline shift often prompt surgical evacuation with a burr hole or craniotomy. However, the recurrence rates of cSDH can reach 37%, and patients with multiple comorbidities requiring antiplatelets and anticoagulants present an even greater challenge for retreatment (5–8).

Many pathological and histological causes, such as inflammatory cytokines, hyperfibrinolysis, and angiogenesis, which lead to neovascularization and repetitive bleeding from fragile capillaries, have been considered responsible for the multiple reaccumulation of hematomas despite surgical evacuation (9–13). Several studies have suggested using pharmacological treatments such as atorvastatin, dexamethasone, and angiotensin-converting enzyme inhibitors to target these pathophysiological factors. Still, the benefits of these therapies for cSDH have remained limited (14–16). After these unsuccessful treatment attempts, embolization of the middle meningeal artery (MMA) has recently been considered to prevent the rebleeding of cSDHs by definitively eliminating neovascularization in the outer membrane. Various case reports and single- or multicenter studies have demonstrated the success of embolization as a minimally invasive technique for treating new, recurrent, and refractory cSDHs (17–22).

In this study, we sought to investigate the safety and effectiveness of MMA embolization for cSDH patients.

## Methods

### Patient selection

A total of 245 patients were diagnosed with cSDH using standard computed tomography (CT) images at our institution from January 2019 to June 2022. We excluded 26 patients whose medical records were not authorized for use. Further, 55 patients who underwent cSDH evacuation concurrently with MMA embolization were also excluded from this study. Of the remaining 164 patients, two patients with unilateral cSDH had an MMA variant originating from the ophthalmic artery. For this reason, MMA embolization was not attempted in these two subjects. A total of 162 patients successfully underwent MMA embolization without combined surgical evacuation for the treatment of new (not previously treated) or recurrent cSDH (previously treated with surgical evacuation) in our institute, but 18 individuals with no imaging 24 h after an embolization were excluded from the study.

**Figure 1:**
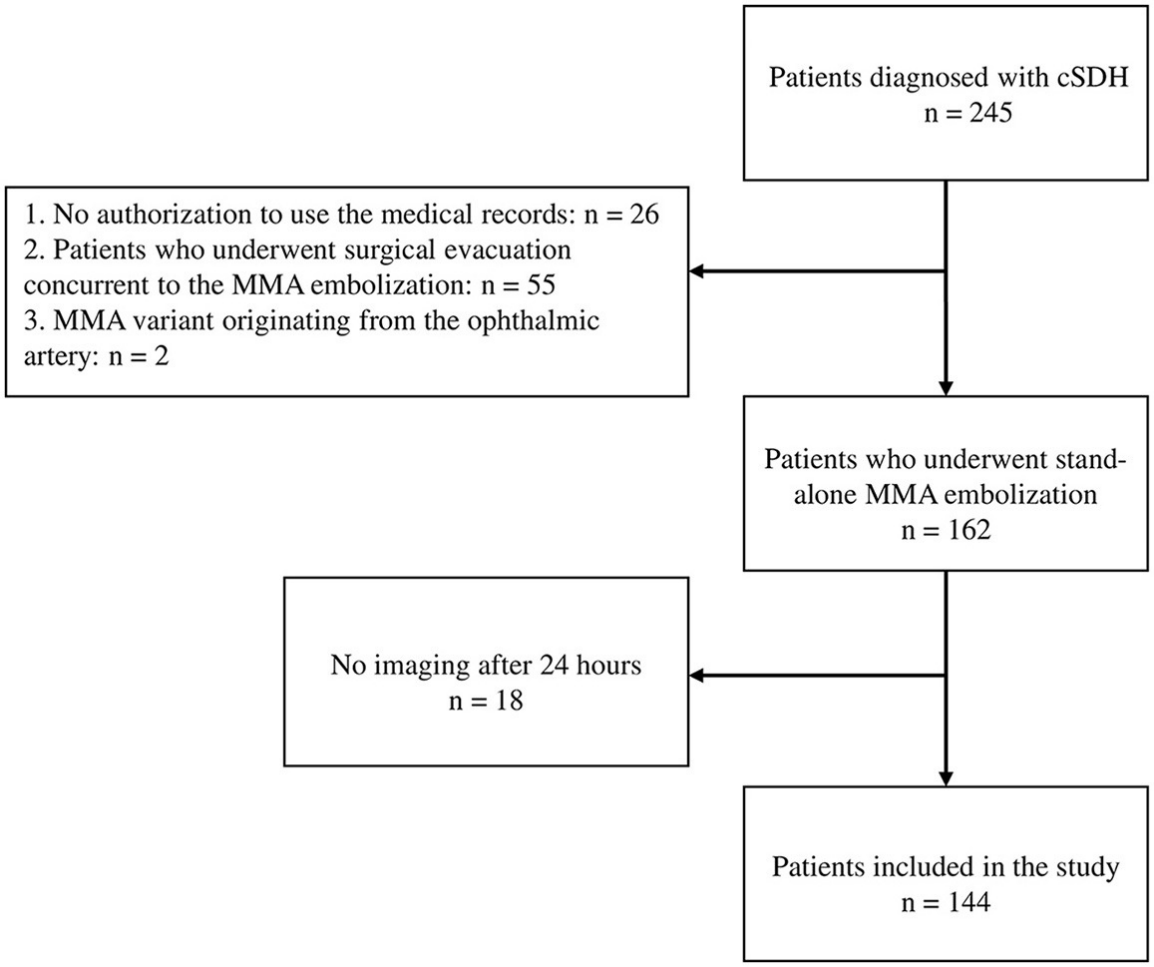
Flowchart for patient selection.

In this study, we present a retrospectively collected database of 144 patients treated with MMA embolization for the treatment of cSDHs as a single center experience from January 2019 to June 2022 (Figure 1). The embolization procedure was discussed with the patients in detail. The inclusion criteria for MMA embolization involved patients with symptomatic cSDH, those with high surgical risk due to comorbidities or advanced age, those with recurrent or refractory hematomas, or those who are poor surgical candidates or have failed conservative management. The exclusion criteria included patients with significant clinical symptoms (worsening mental status, difficulty walking), active bleeding disorders, extensive cerebral edema, a significant midline shift of > 5 mm (MLS, measured as the maximum displacement of the septum pellucidum that crosses the center line of the brain on CT imaging), or those without symptoms or MLS who could be followed conservatively.

### MMA embolization procedure

Informed consent was obtained from all patients before the embolization procedure, and the institutional review board's approval was obtained before collecting the data for this study. Depending on the general clinical condition of the patient, general anesthesia, monitored anesthesia care, conscious sedation, or no sedation was administered during this intervention. The patient was continuously monitored with real-time oxygen saturation, heart rate, ECG rhythm strip, and blood pressure throughout the procedure. Using a sterile technique and ultrasound guidance, a 5-Fr sheath was inserted into the femoral or radial artery using a modified Seldinger technique, and intraarterial heparin was administered. A 5-Fr catheter was navigated into the common carotid artery (CCA) or external carotid artery (ECA), a microcatheter was then navigated over a micro guidewire into the MMA, and selective angiography was performed. If no dangerous collaterals were observed and the ECA angiography demonstrated the origin of the MMA at its usual location from the internal maxillary artery, then the MMA was embolized with the Contour™ PVA Embolization particles (Boston Scientific, Marlborough, Massachusetts, USA), and/or coils in its main trunk before bifurcation and/or selectively in its frontal and parietal branches. Control angiographies of MMA, ECA, and CCA were performed after the embolization.

### Outcome evaluation

In this study, the primary outcome was retreatment for cSDH needing rescue intervention following embolization within 90 days or at the last follow-up. Retreatment was defined as recurrent cSDH with reaccumulation or acute rebleeding on follow-up CT imaging, refractory cSDH with no change in size on interval imaging, or symptomatic cSDH in patients with new or worsening symptoms during clinical follow-ups. Additionally, we performed a subgroup analysis of patients who required retreatment following the embolization procedure. If the patient did not need retreatment for any of these conditions, the MMA embolization procedure was considered successful.

Secondary outcomes were regarded as a >50% reduction in cSDH maximum width (defined as the largest hematoma thickness measured in axial planes) on the last CT imaging, complications, and improvement of the modified Rankin Scale (mRS). The change in the size of cSDH was evaluated on follow-up CT imaging by independent radiologists who were unaware of this study. Complications included adverse events during intraprocedural time or post-procedural clinical or radiological follow-ups. The mRS score for each patient was evaluated based on their neurological disabilities at 90 days or the last clinical follow-up, and changes in the score were reported.

### Statistical analysis

To analyze the maximum width of cSDHs before and after embolization at different follow-up times, e.g., 24 h, 2 weeks, 6 weeks, 90 days, greater than 90 days, and the last follow-up, a statistical analysis was performed using SPSS Statistics software (Version 29, IBM Corporation, New York, USA), and several statistical tests were employed to assess the results. The normality of the data was tested using the Shapiro–Wilk test for a sample size of < 50 and the Kolmogorov-Smirnov test for a sample size of >50. The paired sample *t*-test for normally distributed data and the Wilcoxon Signed Rank test for non-normally distributed data were used to determine the significance of the difference in the maximum width of cSDHs before and after embolization at each follow-up time.

## Results

### Patient characteristics

The baseline characteristics of 144 patients [99 men (68.7%) and 45 women (31.3%)] who underwent MMA embolization are presented in Table 1. The mean age at presentation was 71.5 ± 12 years. The criteria for MMA embolization were considered as presenting symptoms, mass effect, midline shift, and risk of using antiplatelets or anticoagulants. The most common symptoms on admission were headache (22.9%), frequent falls (18.7%), altered mental status (6.9%), gait instability (6.9%), and weakness (4.1%). Of all patients undergoing embolization, 122 (84.7%) patients had a mass effect, 83 (57.6%) patients had a midline shift, and 94 (65.2%) patients were on antiplatelet and/or anticoagulant medication on admission. Comorbidities included hypertension (65.2%), hyperlipidemia (59.7%), diabetes mellitus (29.1%), chronic kidney disease (29.1%), atrial fibrillation (25%), heart failure (24%), coronary artery disease (23.6%), malignancy (22.9%), prior stroke/TIA (21.5%), and benign prostatic hyperplasia (20.1%). Furthermore, alcohol use (67.3%) and tobacco use (50.6%) were also noted among the patients. A total of 67 (46.5%) patients were using antiplatelet medication on admission. Of these patients, 56 (38.8%) were on 81 mg aspirin per day, five (3.5%) were on 325 mg aspirin per day, and one (0.6%) was on clopidogrel. Five patients (3.5%) were taking dual antiplatelet medication. Of these, four (2.8%) and one (0.7%) were on 81 mg aspirin and clopidogrel and 81 mg aspirin and ticagrelor, respectively. The mean platelet count on admission was 213± 103 K/μL. In total, 48 patients (33.3%) were on anticoagulant medication on admission. Of these, 28 (19.4%) were on warfarin, 12 (8.3%) were on apixaban, four (2.8%) were on rivaroxaban, and four (2.8%) were on enoxaparin. A total of 19 patients (13.2%) had a history of treatment for unilateral or bilateral SDHs. Of these patients with previous SDH history, 17 (11.8%) had been treated with burr hole drainage, one (0.7%) had been treated with craniotomy evacuation, and one (0.7%) underwent MMA embolization.

**Table 1:** General characteristics of 144 patients who underwent MMA embolization for cSDHs.

**Patient characteristics**	**Number (percentage), *N =* 144**
**Demographic**	
Age, years (mean, SD)	71.5 ± 12
Men	99 (68.7)
**Ethnicity**	
White patients	137 (95.1)
Asian patients	5 (3.4)
Other	2 (1.4)
**Criteria for MMA embolization**	
Presenting symptoms	
Headache	33 (22.9)
Altered mental status	10 (6.9)
Dizziness	4 (2.7)
Frequent falls	27 (18.7)
Gait instability	10 (6.9)
Weakness	6 (4.1)
Upper extremity numbness	4 (2.7)
Aphasia	3 (2.0)
Dysarthria	3 (2.0)
Memory difficulties	1 (0.6)
Seizure	2 (1.4)
Mass effect	122 (84.7)
Midline shift	83 (57.6)
Risk of antiplatelet or anticoagulant use	94 (65.2)
**Comorbidities**	
Hypertension	94 (65.2)
Hyperlipidemia	86 (59.7)
Dyslipidemia	9 (6.2)
Diabetes mellitus	42 (29.1)
Obesity	35 (24.3)
Heart failure	35 (24.3)
Coronary artery disease	34 (23.6)
Valvular heart disease	14 (9.7)
Atrial fibrillation	36 (25.0)
Prior DVT/PE	18 (12.5)
Prior stroke/TIA	31 (21.5)
Chronic kidney disease	42 (29.1)
Benign prostatic hyperplasia	29 (20.1)
Primary brain tumor	4 (2.7)
Malignancy	33 (22.9)
Irritable bowel syndrome	6 (4.1)
Glaucoma	9 (6.2)
Thrombocytopenia	16 (11.1)
Pancytopenia	6 (4.1)
Depression	20 (13.8)
Dementia	6 (4.1)
Parkinson's disease	5 (3.4)
Current or past alcohol use	97 (67.3)
Current or past tobacco use	73 (50.6)
Antiplatelet medication	67 (46.5)
Aspirin 81 mg	56 (38.8)
Aspirin 325 mg	5 (3.4)
Clopidogrel	1 (0.6)
Aspirin 81 mg + Clopidogrel	4 (2.7)
Aspirin 81 mg + Ticagrelor	1 (0.6)
Admission platelet count (mean, SD)	213 ± 103
Anticoagulation medication	48 (33.3)
Warfarin	28 (19.4)
Apixaban	12 (8.3)
Rivaroxaban	4 (2.7)
Enoxaparin	4 (2.7)
Previous SDH	19 (13.1)
Previous SDH treatment	19 (13.1)
Evacuation	18 (12.5)
Burr hole	17 (11.8)
Craniotomy	1 (0.6)
Embolization	1 (0.6)

SD, standard deviation; MMA, middle meningeal artery; DVT, deep vein thrombosis; PE, pulmonary embolism; TIA, transient ischemic attack; SDH, subdural hematoma.

### cSDH characteristics

Table 2 shows the main characteristics of a total of 220 cSDHs diagnosed in 144 patients. Of these hematomas, 194 (88.1%) were previously untreated new hematomas, 26 (11.8%) were recurrent hematomas despite previous interventions, 150 (68.2%) were bilateral, 37 (16.8%) were only left-sided, and 33 (15%) were only right-sided. cSDHs were found in four different locations, namely, the holohemispheric region covering the entire convexity of the brain in 15 (6.8%), the frontoparietal region in 193 (87.7%), only the frontal region in 11 (5%), and the parietooccipital region in one (0.5%). At admission, the median [interquartile range (IQR) identified as the 25th and 75th percentiles] maximum width of the hematomas and MLS were 12 (8, 15.5) mm and 2 (0,5) mm, respectively. Membranes were seen on CT imaging in 207 cSDHs (94.1%).

**Table 2:** General characteristics of 220 cSDHs.

**SDH characteristics**	**Number (Percentage), *N =* 220**
New SDH (Previously untreated)	194 (88.1)
Recurrent SDH (After failed treatment)	26 (11.8)
**Laterality**	
Bilateral	150 (68.2)
Left only	37 (16.8)
Right only	33 (15.0)
**Location**	
Holohemispheric	15 (6.8)
Frontoparietal	193 (87.7)
Frontal only	11 (5.0)
Parietooccipital	1 (0.5)
Maximum SDH width at admission, mm (median, IQR)	12 (8,15.5)
Midline shift, mm (median, IQR)	2 (0,5)
Membrane	207 (94.1)

SDH, subdural hematoma; mm: millimeters; IQR, interquartile range.

### MMA embolization procedure details

Unilateral MMA embolization in 14 patients with bilateral cSDHs could not be performed because of the following reasons: treatment of 8 unilateral cSDHs with MMA embolization was not preferred by doctors because it was not noticeably thicker than previous non-contrast CT scans; 4 MMAs were not embolized due to their origin from the ophthalmic artery; and 2 MMA embolizations were not performed because satisfactory catheter positioning could not be achieved due to tortuosity of the brachiocephalic artery and aorta. Three patients with unilateral cSDHs underwent bilateral MMA embolization because contralateral MMA supplied the dura in the hematoma region. Thus, a total of 209 MMA embolization procedures were successfully performed for 220 cSDHs in 144 patients. The procedure details are summarized in Table 3. Radial access was used in 125 cases (59.8%). Conscious sedation and procedural heparin were administered in 115 (55%) and 190 cases (90.9), respectively. The mean intraprocedural duration was 30.6± 14.5 mm. The median (IQR) size of MMA was 1 (0.9, 1) mm. Dangerous collaterals (orbital, meningolacrimal, and petrous branches) were determined in 20 cases (9.6%) before the embolization procedure. Following selective microcatheter angiography, embolization was performed using 150–250 micron PVA particles in 204 cases (97.6%), 250–350 micron PVA particles in 1 case (0.5%), 150–250 and 350–500 micron PVA particles in 1 case (0.5%), 150–250 and 355–500 micron PVA particles in 1 case (0.5%), and 150-250 micron PVA particle and coil in 2 cases (0.9%). Under roadmap navigation and continuous fluoroscopy, contour PVA particles were injected until there was a notable decrease in flow through the distal MMA branches. Based on hematoma location and dangerous collateral, the catheter was placed in the main trunk before the bifurcation in 193 cases (92.3%), in the main trunk before the bifurcation and petrous branch in 1 case (0.5%), in both the frontal and parietal branches separately in 12 cases (5.8%), and in the frontal branch only in three cases (1.4%)before embolization.

**Table 3:** Details of the 209 MMA embolization procedure.

**Embolization procedural details**	**Number (Percentage), *N =* 209**
Femoral access	84 (40.2)
Radial access	125 (59.8)
Anesthesia	12 (5.8)
**General anesthesia**	
MAC	13 (6.2)
CS	115 (55)
No sedation	69 (33)
Procedural heparin	190 (90.9)
Procedure duration, min (mean, SD)	30.6 ± 14.5
MMA size, mm (median, IQR)	1 (0.9,1)
Dangerous collaterals seen	20 (9.6)
**Embolization materials**	
150–250 micron PVA particles only	204 (97.6)
250–350 micron PVA particles only	1 (0.5)
150–250 and 350–500 micron PVA particles	1 (0.5)
150–250 and 355–500 micron PVA particles	1 (0.5)
150–250 micron PVA particles & Coil	2 (0.9)
**Catheter position before embolization**	
Main MMA trunk before bifurcation	193 (92.3)
Main MMA trunk before bifurcation and petrous branch	1 (0.5)
Frontal and parietal branches	12 (5.8)
Frontal branch only	3 (1.4)

MAC, monitored anesthesia care; CS, conscious sedation; min, minutes; SD, standard deviation; MMA, middle meningeal artery; mm, millimeters; IQR, interquartile range; PVA, polyvinyl alcohol.

### Clinical outcomes

Table 4 details the clinical follow-up outcomes for 144 patients. The median (IQR) length of hospital stay was 2 (0,9) days. The mean follow-up was 107.2 ± 84.9 days. One patient (0.7%) reported pain out of proportion to the procedure, where a parietal branch of MMA was embolized with 150-250 PVA particles. Control angiography was performed and demonstrated some shunting of contrast into the transverse sinus through a small, iatrogenic fistula. This fistula was caused by increased hydrostatic pressure within the vessel through the occlusive microcatheter. This branch was further embolized with 350–500 PVA particles, and control angiography confirmed the occlusion of the parietal branch. The patient's headache gradually improved, and the subject tolerated the procedure well and remained neurologically unchanged throughout the procedure. No other symptoms related to this complication developed during follow-up. Three patients (2.1%) experienced postprocedural complications, such as new mixed aphasia without motor deficits, intermittent episodes of left-sided weakness, and seizures. Access site complications were not observed. A total of 12 patients (8.4%) died from causes unrelated to the procedure during follow-up. The mean (SD) mRS scores on admission, 90-day follow-up, and improvement were 2.3 ± 1.2, 1.7 ± 1.6, and 0.7 ± 0.8, respectively. Improvement in mRS scores was noticed in 71 patients (49.3%), and worsening was noticed in 11 patients (7.6%). There were 76 patients (52.8%) on admission and 99 patients (68.8%) at 90-day follow-up with an mRS of ≤ 2, with a relative ratio of 1:1.3, and there were 68 patients on admission and 35 patients (24.3%) at 90-day follow-up with an mRS score between 3 and 5, with a relative ratio of 2:1.

**Table 4:** Clinical follow-up results for 144 patients who underwent MMA embolization for the treatment of cSDH.

**Clinical outcomes**	**Number (Percentage), *N =* 144**
Length of hospital stay, days (median, IQR)	2 (0,9)
Follow-up, days (mean, SD)	107.2 ± 84.9
Complications	4 (2.8)
Intraprocedural complications	1 (0.7)
Iatrogenic fistula	1 (0.7)
Postprocedural complications	3 (2.1)
Aphasia	1 (0.7)
Weakness	1 (0.7)
Seizure	1 (0.7)
Need for retreatment	20 (13.8)
Mortality	12 (8.4)
Causes of death	
Malignancy	2 (1.4)
End-stage renal disease	2 (1.4)
End-stage liver disease	2 (1.4)
Graft vs. Host Disease	1 (0.7)
Dementia	1 (0.7)
Acute respiratory failure	1 (0.7)
Toxic shock syndrome	1 (0.7)
Neutropenic fever	1 (0.7)
Multiple organ failure	1 (0.7)
mRS score	
At presentation (mean, SD)	2.3 ± 1.2
0–2	76 (52.8)
3–5	68 (47.2)
At 90-day follow (mean, SD)	1.7 ± 1.6
0–2	99 (68.8)
3–5	35 (24.3)
6 only	10 (6.9)
Improvement (mean, SD)	0.7 ± 0.8
Improvement	71 (49.3)
No difference	62 (43.1)
Worsening	11 (7.6)

IQR, interquartile range; SD, standard deviation; mRS, modified Rankin Scale score.

### Subgroup analysis

A total of 20 patients (13.8%) required retreatment of the hematoma after the MMA embolization procedure. Table 5 presents the results of the subgroup analysis of factors associated with hematoma recurrence. In total, 14 patients (70%) were re-treated with a burr hole (13/14) or craniotomy (1/14) during the follow-up period due to the increased size of the recurrent hematomas after embolization. The embolization procedure was repeated for three patients (15%) due to refractory hematomas that did not sufficiently decrease in size despite previous embolization. Three patients (15%) required surgical rescue for symptomatic hematomas due to the development of new or worsening symptoms without an increase in the size of the hematoma. One day after embolization, persistent seizure activity and tenuous respiratory status on non-invasive therapy were observed in one patient, after which a decision was made to proceed with intubation, and the patient underwent craniotomy evacuation. Another patient was admitted with a chief complaint of acute dysarthria six months after the embolization procedure, and a burr hole evacuation was performed. One more patient presented with a complaint of headache described as “pressure” for two days, and the patient underwent burr hole evacuation.

**Table 5:** Subgroup analysis of 20 patients who required retreatment of the hematoma after the MMA embolization procedure.

**Subgroup analysis**	**Number (Percentage), *N =* 20**
**Reasons for retreatment**	
Recurrence of SDH	14 (70)
Repeat burr hole	13 (65)
Repeat craniotomy	1 (5)
Refractory SDH	3 (15)
Repeat embolization	3 (15)
Symptomatic SDH	3 (15)
Repeat burr hole	2 (10)
Repeat craniotomy	1 (5)
Antiplatelet medication	10 (50)
Aspirin 81 mg	8 (40)
Aspirin 325 mg	2 (10)
Anticoagulation medication	7 (35)
Warfarin	2 (10)
Apixaban	3 (15)
Rivaroxaban	2 (10)
**Comorbidities**	
Hypertension	15 (75)
Hyperlipidemia	14 (70)
Diabetes mellitus	6 (30)
Heart failure	6 (30)
Coronary artery disease	6 (30)
Valvular heart disease	3 (15)
Atrial fibrillation	4 (20)
Prior DVT/PE	5 (25)
Prior stroke/TIA	4 (20)
Thrombocytopenia	4 (20)
Current or past alcohol use	10 (50)
Current or past tobacco use	10 (50)

SDH, subdural hematoma; DVT, deep vein thrombosis; PE, pulmonary embolism; TIA, transient ischemic attack.

Upon admission, 10 patients (50%) were utilizing antiplatelet medications. Among them, eight patients (40%) were taking low-dose aspirin (81 mg per day), and two patients (10%) were taking high-dose aspirin (325 mg per day). Similarly, seven patients (35%) were under anticoagulation treatment upon admission. Among these seven patients, two (10%) were on warfarin, three (15%) were on apixaban, and two (10%) were on rivaroxaban. The most common comorbidities included hypertension (75%), hyperlipidemia (70%), diabetes mellitus (30%), heart failure (30%), coronary artery disease (30%), prior DVT/PE (25%), atrial fibrillation (20%), prior stroke/TIA (20%), thrombocytopenia (20%), and valvular heart disease (15%). Furthermore, 50% of the patients reported using alcohol, and 50% of them reported using tobacco.

### Radiographic outcomes

**Figure 2:**
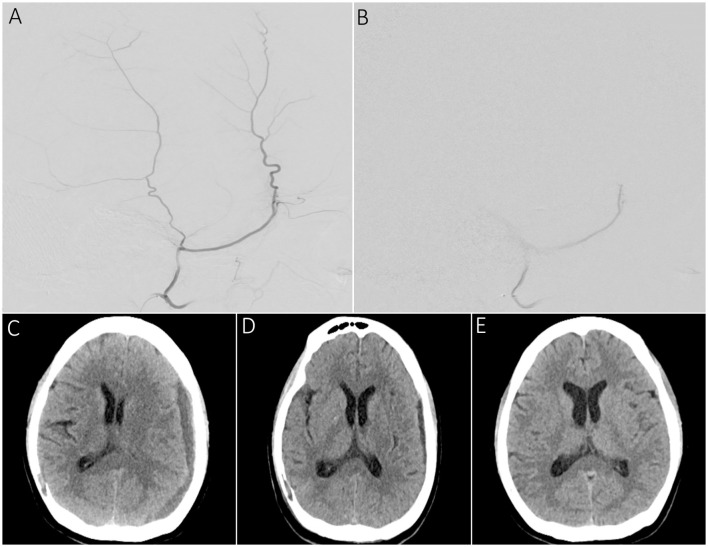
A sample patient presenting with dysarthria and right-hand tingling undergoing MMA embolization for chronic SDH. **(A)** Supra-selective lateral angiography showing the frontal and parietal branches of the MMA before the embolization procedure. **(B)** Supra-selective MMA angiography demonstrating persistent proximal main trunk occlusion following PVA particle embolization. **(C)** Pre-embolization CT scan showing a left convex cSDH with a maximum width of 12 mm and a midline shift of 5 mm. **(D)** 2-week follow-up scan showing a reduction in maximum width of the SDH up to 6 mm and resolution of prior midline shift. **(E)** 3-month follow-up scan demonstrating complete resolution of cSDH.

Of the 220 cSDHs, 14 could not be treated with embolization for the abovementioned reasons. Table 6 shows the radiographic outcomes for embolized 206 cSDHs in 144 patients. The median (IQR) maximum width of 206 cSDHs at pre-intervention and the last follow-up were 12 mm (8, 16) and 3 mm (0.6), respectively. The median maximum width reduction from admission and reduction percentage was 8 mm (4, 13) and 77.5% (43%,100%), respectively. Of the 206 cSDHs, the mean (SD) maximum width follow-up was 109.6 ± 85.8 days, where 24 (11.7%) had a follow-up of 2 weeks, 42 (20.4%) had a follow-up of 6 weeks, 57 (27.7%) had a 90-day follow-up, and 83 (40.3%) had a follow-up of > 90 days. At the longest follow-up, the maximum width improved in 194 cSDHs (94.2%) and worsened in two cSDHs (1%). At least 20% improvement was observed in 188 (91.2%), 50% improvement in 150 (72.8%), 70% improvement in 118 (57.2%), and complete resolution in 90 (43.6%) patients with cSDH (Figure 2). Table 7 shows the improvement in the mean (SD) cSDHs maximum width from 24 h to the last follow-up during each follow-up period. Based on the results of the statistical analysis performed on the maximum width of cSDHs before and after embolization, it was found that there was a statistically significant reduction in the maximum width of cSDHs over time starting from the 2-week follow-up period of embolization, with a significance level of a *p*-value of < .001. These results suggest that MMA embolization was effective in reducing the maximum width of cSDHs over time.

**Table 6:** Radiographic follow-up results for 206 cSDHs treated with MMA embolization.

**Radiographic outcomes**	**Number (Percentage), *N =* 206**
**Max SDH width**	
Pre-intervention, mm (median, IQR)	12 (8, 16)
At the last follow-up, mm (median, IQR)	3 (0,6)
Max width reduction from admission (median, IQR)	8 (4, 13)
Reduction percentage (median, IQR)	77.5% (43%,100%)
**SDH max width follow-up**	
2 weeks	24 (11.7)
6 weeks	42 (20.4)
90 days	57 (27.7)
> 90 days	83 (40.3)
Follow-up, days (mean, SD)	109.6 ± 85.8
**Max SDH width improvement**	
Improvement	194 (94.2)
No difference	10 (4.8)
Worsening	2 (1)
**Degree of max SDH width of reduction**	
>20% reduction	188 (91.2)
>50% reduction	150 (72.8)
>70% reduction	118 (57.2)
100% reduction	90 (43.6)

SDH, subdural hematoma; max: maximum; mm, millimeters; IQR, interquartile range; SD, standard deviation.

**Table 7:** Improvement in the maximum width of 206 cSDHs during follow-up.

	**max SDH width, mm**			
**Follow-up time**	**Pre-embolization (Mean, SD)**	**Post-embolization (Mean, SD)**	**Number of procedures**	***p*** **-value**
24 h	12.6 ± 5.4	12.3 ± 5.3	37	0.146
2 weeks	12.6 ± 5.5	10.9 ± 6.6	138	< 0.001
6 weeks	12.7 ± 5.3	8.0 ± 6.4	142	< 0.001
90 days	12.7 ± 5.4	5.6 ± 5.8	106	< 0.001
>90 days	13.1 ± 5.4	3.7 ± 4.9	73	< 0.001
Last follow-up	12.4 ± 5.3	4.1 ± 5.0	206	< 0.001

SD, standard deviation; max, maximum; mm, millimeters.

## Discussion

In this study, we present several important clinical and radiological findings. First, MMA embolization provided at least a 50% reduction in the maximum width of cSDH in 72.8% of 144 patients. Second, 13.8% of patients needed retreatment for recurrent, refractory, or symptomatic cSDHs — a total of 23 cSDHs (11.2%). Third, one patient (0.7%) experienced an intraprocedural complication without other symptoms related to this complication during the follow-up period, and three (2.1%) patients experienced postprocedural complications. Fourth, the preoperative mRS score improved in 49.3% of patients, with a mean (SD) improvement score of 0.7 (0.8) at the 90-day follow-up. These findings are substantial because they demonstrate that MMA embolization alone can provide a significant reduction in the size of cSDH with relatively low recurrence and complication rates. However, it is essential to emphasize that MMA embolization should not be presented as a substitute for surgical evacuation but rather as a viable alternative for select patients who may benefit from a less invasive approach. Careful consideration of the inclusion and exclusion criteria ensures appropriate patient selection and aids in optimizing treatment outcomes in the management of cSDH.

Embolization treatment plays a crucial role in the management of cSDH, particularly in challenging patient populations. In determining the specific population of cSDH patients who are likely to benefit from MMA embolization, our analysis highlights a few distinct patient groups. These groups encompass patients at high risk of recurrence with comorbidities such as cardiovascular disease or alcohol use, patients who are receiving antithrombotic therapy or those with persistently low platelet counts warranting intervention to manage their condition effectively, and patients presenting with minor symptoms such as isolated headache or gait instability but still requiring medical intervention. In our study, we also identified several potential risk factors for hematoma recurrence. Of the 20 patients who needed retreatment, 17 were on antiplatelet and/or anticoagulant medication. The recurrence rates were higher in patients with comorbidities such as cardiovascular disease, impaired coagulation profiles, chronic alcoholism, and liver cirrhosis. Our study did not investigate the type of cSDH that may have impacted the rates of recurrence (23, 24). Future studies should seek to further assess the relationship between this subset of cSDH and outcomes. Additionally, previous literature has documented a rare complication of cerebral abscess, which was not observed in our study (25).

A propensity-adjusted analysis by Catapano et al. (26) found fewer treatment failures and more significant reductions in hematoma volume in the embolization group compared to conventional management, which includes surgical evacuation and conservative therapy. A meta-analysis by Ironside et al. (27) showed that embolization had lower rates of recurrent cSDHs and surgical rescue rates than conventional treatment in five studies with a total of 902 patients. With accumulating positive evidence, MMA embolization alone is increasingly being utilized in clinical settings for carefully selected patients. In a prospective multi-center case series, Kan et al. (21) achieved outcomes similar to our study, with at least a 50% improvement in SDH thickness in 70.8% of cases and an improvement in mRS score in 31.9% of patients with MMA embolization alone. Our cSDH thickness improvement rate was comparable to the rate found in their study. However, in our study, improvement in the mRS score reached 50%. Despite our slightly higher retreatment rate, which might be explained by the fact that ~40% of our patient population was taking antithrombotic medications. Additionally, our patient population was notably older compared to the Kan et al. (21) cohort. Several studies have documented that the risk of hematoma recurrence and complications is higher in older adults or comorbid patients who take antithrombotic agents (22, 28).

Patients should be thoroughly evaluated for potential anastomoses and anatomical variations of MMA before an embolization is performed. Fantoni et al. (29) reported that patients with cSDHs had a much higher prevalence of MMA originating from the ophthalmic artery in cSDHs when compared with the control group of patients undergoing embolization for epistaxis. In our case series, internal carotid artery cerebral angiography also confirmed that 4 MMA arose as a recurrent branch of the ophthalmic artery. In addition to anatomical variation, the anterior or petrosal branch of the MMA can anastomose to the ophthalmic artery, internal carotid artery, or vertebral artery, and the petrosal branch can also supply the vasa nervorum of the facial nerve (30–32). Kan et al. (21) reported a patient in whom embolization material escaped into the vasa nervorum of the facial nerve. In our study, the petrosal branch of MMA was embolized in only one patient because it was determined to provide most of the hematoma. Before embolization, super-selective microcatheter angiography demonstrated that the petrous branch did not anastomose with the internal carotid artery and did not supply the vasa nervorum of the facial nerve. No procedure-related complication was observed in the patient following the embolization of this branch. When these dangerous collateral branches are observed in the selective angiography, embolization must be performed from a location distal to the origin of the collateral branch in order to prevent ischemic complications (33).

Our study is limited by its retrospective nature. A standard protocol was not applied for the treatment of cSDH in patients. However, our institute used a particular follow-up image protocol to monitor the change in the maximum width of cSDHs. Although the treatment options were discussed with the patients, the attending neurosurgeons decided whether the patient would receive treatment and the type of treatment, which may cause bias in our outcomes. Finally, this study is limited by its single-arm nature. Nevertheless, the present study demonstrated similar successful results with this technique.

Despite the growing interest in MMA embolization as a minimally invasive treatment option for cSDH, the available literature is limited, with few large-scale, well-designed studies investigating the effectiveness and safety of MMA embolization. To fill this knowledge gap, it is necessary to conduct additional studies, such as randomized controlled trials with long-term follow-up, to provide more robust evidence on the continued safety and efficacy of MMA embolization.

## Conclusion

Our results indicate that MMA embolization alone is a safe and effective minimally invasive technique for the treatment of cSDHs in patients who do not require immediate surgical evacuation. However, it is essential to acknowledge that this approach may not be universally applicable in certain cases due to specific contraindications such as severe renal failure, a lack of suitable access routes to the MMA, or a history of known allergies or hypersensitivity to embolic agents used during the procedure. The identification of contraindications and limitations associated with this technique becomes paramount in ensuring patient safety and optimizing treatment efficacy. There are currently several ongoing trials on MMA embolization, and their results may aid in evidence-based patient selection and further improve the outcomes of MMA embolization.

## Data availability statement

The original contributions presented in the study are included in the article/supplementary material, further inquiries can be directed to the corresponding author.

## Ethics statement

The studies involving humans were approved by the IRB: 19-011249 Outcomes of Medical and Surgical Management of Chronic Subdural Hematomas. The studies were conducted in accordance with the local legislation and institutional requirements. Written informed consent for participation in this study was provided by the participants' legal guardians/next of kin. Written informed consent was obtained from the individual(s) for the publication of any potentially identifiable images or data included in this article.

## Author contributions

AO, YS, CB, and HK contributed to conception and design of the study. AO organized the database and wrote all sections of the manuscript. CB performed the statistical analysis. All authors contributed to manuscript revision, read, and approved the submitted version.
